# Haplotype analysis of sucrose synthase gene family in three *Saccharum* species

**DOI:** 10.1186/1471-2164-14-314

**Published:** 2013-05-10

**Authors:** Jisen Zhang, Jie Arro, Youqiang Chen, Ray Ming

**Affiliations:** 1 College of Life Sciences, Fujian Normal University, Fuzhou 350108, China; 2 Department of Plant Biology, University of Illinois at Urbana-Champaign, Urbana, IL 61801, USA

**Keywords:** Sucrose synthase, Haplotype, Single nucleotide polymorphisms, *Saccharum officinarum*, *Saccharum spontaneum*, *Saccharum robustum*

## Abstract

**Background:**

Sugarcane is an economically important crop contributing about 80% and 40% to the world sugar and ethanol production, respectively. The complicated genetics consequential to its complex polyploid genome, however, have impeded efforts to improve sugar yield and related important agronomic traits. Modern sugarcane cultivars are complex hybrids derived mainly from crosses among its progenitor species, *S. officinarum* and *S. spontanuem*, and to a lesser degree, *S. robustom*. Atypical of higher plants, sugarcane stores its photoassimilates as sucrose rather than as starch in its parenchymous stalk cells. In the sugar biosynthesis pathway, sucrose synthase (*SuSy*, UDP-glucose: D-fructose 2-a-D-glucosyltransferase, EC 2.4.1.13) is a key enzyme in the regulation of sucrose accumulation and partitioning by catalyzing the reversible conversion of sucrose and UDP into UDP-glucose and fructose. However, little is known about the sugarcane *SuSy* gene family members and hence no definitive studies have been reported regarding allelic diversity of *SuSy* gene families in *Saccharum* species.

**Results:**

We identified and characterized a total of five sucrose synthase genes in the three sugarcane progenitor species through gene annotation and PCR haplotype analysis by analyzing 70 to 119 PCR fragments amplified from intron-containing target regions. We detected all but one (i.e. *ScSuSy5)* of *ScSuSy* transcripts in five tissue types of three *Saccharum* species. The average SNP frequency was one SNP per 108 bp, 81 bp, and 72 bp in *S. officinarum, S. robustom*, and *S. spontanuem* respectively. The average shared SNP is 15 between S*. officinarum* and *S. robustom*, 7 between *S. officinarum* and *S. spontanuem* , and 11 between *S. robustom* and *S. spontanuem*. We identified 27, 35, and 32 haplotypes from the five *ScSuSy* genes in *S. officinarum, S. robustom*, and *S. spontanuem* respectively. Also, 12, 11, and 9 protein sequences were translated from the haplotypes in *S. officinarum, S. robustom, S. spontanuem*, respectively. Phylogenetic analysis showed three separate clusters composed of *SbSuSy1* and *SbSuSy2*, *SbSuSy3* and *SbSuSy5*, and *SbSuSy4*.

**Conclusions:**

The five members of the *SuSy* gene family evolved before the divergence of the genera in the tribe Andropogoneae at least 12 MYA. Each *ScSuSy* gene showed at least one non-synonymous substitution in SNP haplotypes. The SNP frequency is the lowest in *S. officinarum*, intermediate in *S. robustum*, and the highest in *S. spontaneum*, which may reflect the timing of the two rounds of whole genome duplication in these octoploids. The higher rate of shared SNP frequency between *S. officinarum* and *S. robustum* than between *S. officinarum* and in *S. spontaneum* confirmed that the speciation event separating *S. officinarum* and *S. robustum* occurred after their common ancestor diverged from *S. spontaneum*. The SNP and haplotype frequencies in three *Saccharum* species provide fundamental information for designing strategies to sequence these autopolyploid genomes.

## Background

Sugarcane (*Saccharum* spp.) is an agronomically important grass that contributes about 80% of the world sugar production (FAOSTAT, 2010) and, more recently, has become a major biofuel feedstock, contributing about 40% of ethanol production worldwide [1]. Consequently, sugarcane breeding efforts is now largely geared towards improvement in both sugar and biomass yield.

Although considerable improvement has been made in sugar yield in the past decades, sugarcane is substantially lagging behind most crops in maximizing gains through molecular breeding. Most of the basic molecular genetic analyses remains unresolved in sugarcane due to unique challenges and complications brought about by a genome with an extreme autoploidy level that can range from octoploidy (x = 8) to dodecaploidy (x = 12). The saccharum complex have no known diploid member species but are all polyploids. *S. officinarum*’s chromosome number is constant at 2n = 80 while that for *S. spontaneum* and *S*. *robustum* ranges from 2n = 36 -128 and 2n = 60 - 160, respectively [2]. *S. spontaneum* have a basic chromosome number x = 8 while both *S. officinarum* and *S*. *robustum* would have x = 10 [3]. Modern sugarcane cultivars are complex autopolyploid and aneuploids of interspecific hybrids derived from *S. officinarum, S. spontaneum* and *S*. *robustum*. About 80-90% of modern day cultivars’ chromosomes are derived from *S. officinarum* and the remaining 10-20% are derived from *S. spontaneum,* and inter-specific recombination [4-6]. Hybrid cultivars’ high sugar content trait is contributed by *S. officinarum*, while the stress tolerance and pest and disease resistance is attributed by *S. spontaneum*. More recently, another well-known vigorous growing wild species, *S. robustum,* is being tapped in some sugarcane breeding programs for enhanced biomass yield.

Due to its high degree of polyploidy and heterozygosity, sequencing the sugarcane genome using the current short-read sequencing technology remains a formidable challenge. For the most part, expressed sequence tags (EST) resources have been the sole resource for sugarcane gene and gene family discovery [7,8]. The recent sequencing and annotation of sorghum bicolor’s genome, the closest diploid relative of sugarcane in the Andropogonae tribe, has served as an indispensable resource for sugarcane genomic studies [9]. Sorghum’s genome size of about 730 Mb [9] is roughly similar to the monoploid genome size of *S. spontaneum* of approximately 843 Mb[3]. The high degree of synthenic collinearity that has been reported by linkage mapping [10-12] and sequence comparison of selected sugarcane bacterial artificial chromosomes (BACs) [9,13,14] have provided some resolution on the complex genetics and inheritance of sugarcane.

Understandably, because sugarcane is grown largely for its sugar and its sugar-derived products like ethanol, gene families related to sucrose metabolism are of paramount importance and are the subject of rigorous molecular genetics interest. Sucrose synthase (*SuSy*, UDP-glucose: Ds-fructose 2-a-D-glucosyltransferase, EC 2.4.1.13) is a major enzyme involved in sucrose metabolism [15-18] and partitioning [19] and is particularly important due to the unique ability of sugarcane to store its photoassimilates in the form of sucrose in its stalks [19-21]. A small multigene family has been found to encode several *SuSy* isoforms in many plant species including maize [22,23], rice [24], Arabidopsis [25] and some other model plant organisms[26,27]. However, aside from an expression analysis of a sugarcane *SuSy* cDNA [21] and a survey in sugarcane EST library, which revealed four *SuSy* clones highly homologous to *SuSy* isoform I [28], little is known about the variation in haplotypes of genes within and among *Saccaharum* species. Due to the complexity of the genome and the lack of whole genome sequence of sugarcane, studies dealing with haplotype analysis of gene families have received little attention.

Previously, the haplotypes of sucrose phosphate synthase III gene were surveyed to examine the association between SNP frequency and sucrose content in sugarcane and its progeny [29]. Haplotype sequences were analyzed for a target genomic region containing a brown rust resistance gene *Bru1* in seven BACs from hybrid cultivar R570, and four, two, and two BACs were classified as *S. officinarum*, *S. spontaneum*, and recombinant haplotypes, respectively [30]. These are the only two studies for sugarcane haplotype sequences, and both used commercial hybrid cultivars as experiment materials, Q165 in the first study and R570 in the second. In order to understand the intra- and inter-species allelic variation of such an important gene like *SuSy*, we surveyed the single nucleotide polymorphisms (SNPs) and haplotypes variation in three founding species for modern sugarcane, *S. officinarum (x = 10), S. spontanuem (x = 8), and S. robustom(x = 8)*. We characterized the *SuSy* gene family members, its evolutionary origin, and the haplotype classes in the three *Saccharum* species known to be the progenitor to modern sugarcane.

## Results

### Identification of five *SuSy* genes in sorghum

We used the six well-annotated sucrose synthase genes in *Arabidopsis thaliana* (TAIR database) to find the corresponding homologous sucrose synthase gene family members in *Sorghum bicolor* (referred from here on as *SbSusy*). Of the five homologous *SbSuSy* genes identified, two were not annotated in the sorghum gene database (Phytozome database version 9, http://ftp.jgi-psf.org/pub/compgen/phytozome/v9.0/Sbicolor_v1.4/). The sequences and location of these five annotated *SbSuSy* genes are listed in Table 1.

**Table 1:** **Sequence similarity of *SuSy *gene fragments between *Saccharum *and *Sorghum bicolor***

**Sorghum**			**Sugarcane**		DNA sequences identity**
**Gene name**	**Chromosome position**	**Protein size**	**Gene name**		
SbSusy 1 Sb01g033060	Chr. 1	816	ScSusy 1	*S. officinarum*	94%
				*S. robustum*	94%
				*S. spontaneum*	94%
*SbSusy 2	Chr. 10	837	ScSusy 2	*S. officinarum*	94%
				*S. robustum*	94%
				*S. spontaneum*	94%
SbSusy 3 Sb04g038410	Chr. 4	838	ScSusy 3	*S. officinarum*	96%
				*S. robustum*	96%
				*S. spontaneum*	96%
SbSusy 4 Sb01g035890	Chr. 1	809	ScSusy 4	*S. officinarum*	95%
				*S. robustum*	96%
				*S. spontaneum*	95%
*SbSusy 5	Chr. 10	892	ScSusy 5	*S. officinarum*	95%
				*S. robustum*	95%
				*S. spontaneum*	95%

Notes: *SbSusy2 and SbSusy5 were not annotated in the Sorghum genome*.*

** The sequence identity id between sorghum and sugarcane orthologous genes.

### *SuSy* gene family is comprised of five genes in sugarcane

The *SuSy* genes in both *Arabidopsis thaliana* and *Sorghum bicolor* were subsequently used to annotate and predict the corresponding SuSy gene family members in sugarcane (referred from here on as *ScSusy*) from the available sugarcane EST database (i.e. sugarcane assembled sequence (SAS)) and RNA-seq data generated in our laboratory. Each of the predicted sugarcane *ScSuSy* genes was verified by sequencing the PCR product amplified from genomic DNA samples of the three accessions: LA-Purple *(S. officinarum)*, SES208 (*S. spontaneum)*, and Molokai6081 (*S. robustum)* (Table 1). The amplified PCR fragments showed an average of 95% sequence similarity to sorghum *SuSy* genes. *ScSuSy1* and *ScSuSy2* showed lower sequence similarity with their sorghum counterparts than the other three *ScSuSy* genes (Table 1).

**Figure 1:**
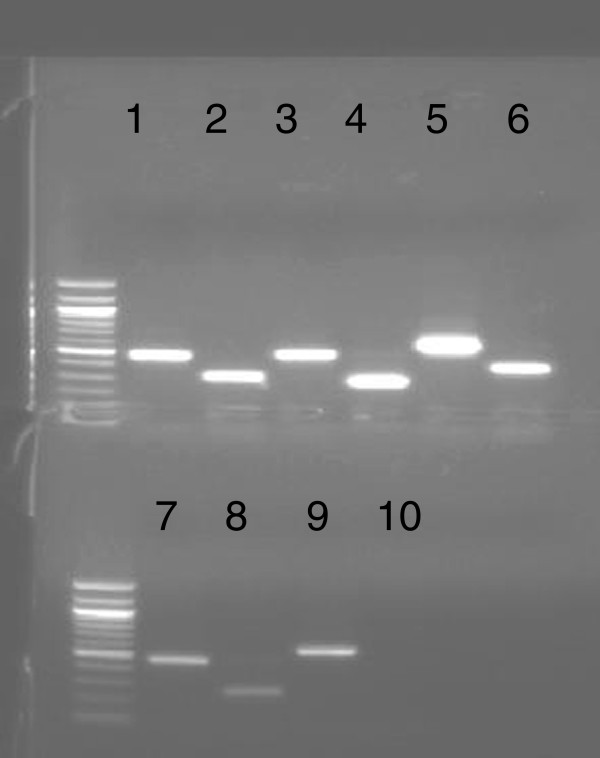
**Results of RT-PCR and genomic PCR amplification of the 5 SuSy genes in *S. o. *(*S. officinarum*).** Lanes 1: Genomic PCR of *SuSy1*; 2:RT-PCR of *SuSy1*; 3: Genomic PCR of *SuSy2*; 4: RT-PCR of *SuSy2*; 5: Genomic PCR of *SuSy3* DNA; 6: RT-PCR of *SuSy3*; 7: Genomic PCR of *SuSy4*; 8 RT-PCR of *SuSy4* ; 9: Genomic PCR of *SuSy5*; 10:RT-PCR of *SuSy5*.

RT-PCR was performed to detect the expression patterns of these five *SuSy* genes for each species in five tissues: leaf roll, mature leaves, the 3rd, 9th, and 15th internode. All except one *(i.e. ScSuSy5)* was consistently detected in all five tissue of each sugarcane species (Figure 1).

We assembled the short-read cDNA sequences for each of the five *ScSuSy* genes derived from RNA-seq analyses of LA Purple leaf tissue (R. Ming, unpublished data). The amino acid sequences were deduced from open reading frames (ORFs) and homology-based analyses (Table 2). The predicted molecular weights of the five polypeptides range from 91.71 to 98.79 kDa while the predicted isoelectric point of the polypeptides range from 5.82 (*ScSuSy2*) to 8.26 (*ScSuSy3*). We found that the predicted amino acid sequences between *ScSuSy1*, *ScSuSy2* and *ScSuSy4* share a consistently higher pairwise sequence similarity (70-80%) in contrast to *ScSuSy3* and *ScSuSy5* (< 70%).

**Table 2:** **Pairwise similarity index between the five predicted *ScSuSy *amino acid sequences in sugarcane**

	**Similarity**				
Gene	*ScSuSy1*	*ScSuSy2*	*ScSuSy3*	*ScSuSy4*	*ScSuSy5*
Protein size	816	802	824	806	866
molecular mass (KDa)	92.96	91.71	93.59	91.95	98.79
Isoelectric point (p*I)*	6.03	5.82	8.26	6.29	6.56
*ScSuSy1*	—	—	—	—	—
*ScSuSy2*	0.80	—	—	—	—
*ScSuSy3*	0.51	0.53	—	—	—
*ScSuSy4*	0.70	0.71	0.54	—	—
*ScSuSy5*	0.54	0.54	0.71	0.55	—

### Phylogenetic analysis of *SuSy* orthologous genes in sugarcane and sorghum

**Figure 2:**
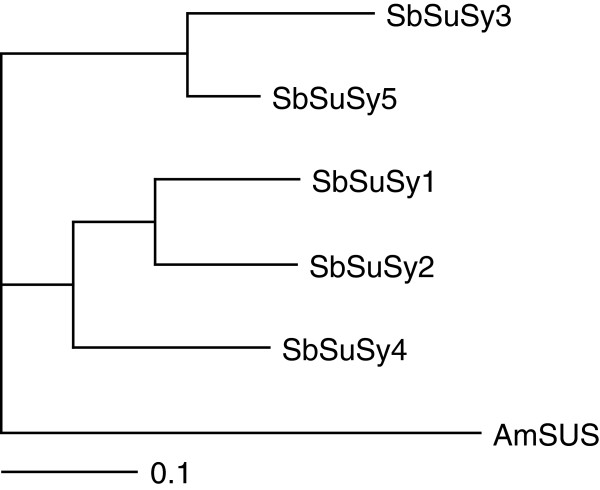
**Phylogenetic relationships between sorghum *sbSuSy *with Anabaena *ASuSy *(a filamentous cyanobacteria) as an outgroup.**

To see the sequence similarity and evolutionary relationship among the *SbSuSy* gene family members in sorghum, an unrooted phylogenetic tree was generated using the full length protein sequences of the *SbSuSy* genes. The phylogenetic tree constructed by the neighbor-joining method formed two well-defined clusters. One cluster contained *SbSuSy3* and *SbSuSy5*, and the other contained *SbSuSy1, SbSuSy2* and *SbSuSy4* (Figure 2).

**Figure 3:**
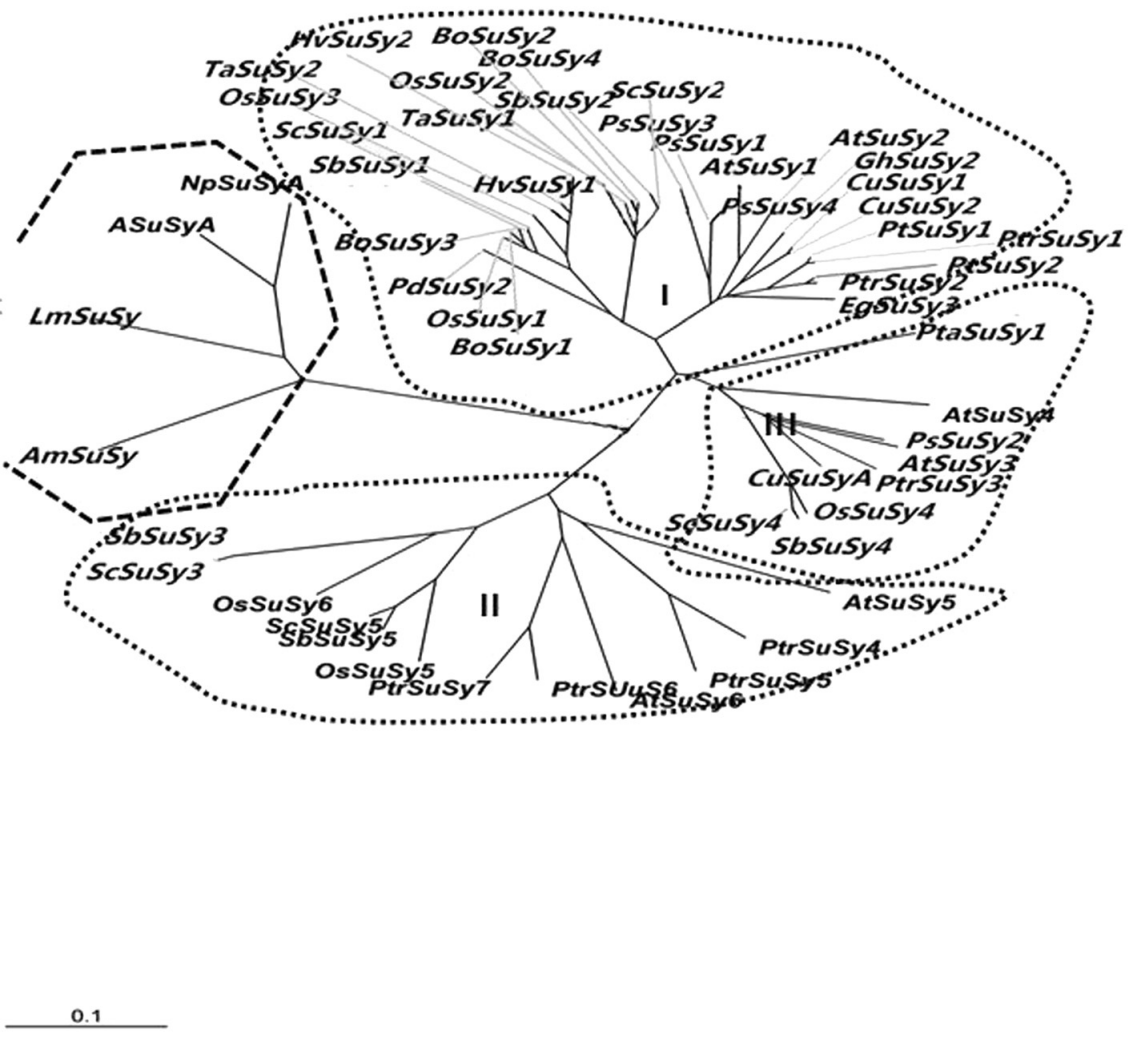
**An unrooted phylogenetic tree derived from *SuSy *protein sequences of sorghum, sugarcane and the other plants (refer to Additional file****1****).**

For comparison, an unrooted phylogenetic tree was likewise constructed for assessing the evolutionary relationship of *SuSy* genes of sorghum and several well-annotated plant and bacterial genomes. Twenty-eight protein sequences from dicots, 26 sequences from monocots, and 4 bacteria sequences were used for constructing the unrooted phylogenetic tree (Additional file 1). All of the bacterial *SuSy* genes clustered into the same group (outgroup), distinctly branching away from the plant *SuSy* gene cluster. The *SuSy* genes of angiosperms could be subdivided into three distinct subgroups, arbitrarily designated as Class I, II and III (Figure 3). *SbSuSy1* and *SbSuSy2*, *SbSuSy3* and *SbSuSy5*, and *SbSuSy4* were distributed in Class I, II and III, respectively (Figure 3). Interestingly, Class I and II seem to reflect the boundary between monocots and dicots.

### Identification of SNPs in the five *ScSuSy* genes within and among *Saccharum* Species

To compare sequence variation and identify single nucleotide polymorphism (SNP) among the five annotated *ScSuSy* genes within and among *Saccharum* species, we designed PCR primers that will amplify a 500 bp region that includes both an exonic and intronic sequences. To reduce the potential confounding issue of intergenomic recombination, SNPs were only reported if found in at least three sequences. To ensure sufficient sequencing depth in octoploids, 70 to 119 amplified fragments were cloned and sequenced per gene per species.

In *ScSuSy1*, four, seven, and eleven single nucleotide polymorphisms (SNPs) were detected within the 489 bp region in *S. officinarum* (LA Purple), *S. robustum* (Molokai6081), and *S. spontaneum* (SES208), respectively. Of the total 22 SNPs, 19 were found within introns. One of these intronic SNPs is consistently present in all three species. In *ScSuSy2*, four, three, and six SNPs were identified within the 484 bp region in the three respective species. In this case, however, none of the SNPs within *ScSuSy*2 are shared in all three species. Of the combined 13 SNPs from the three species, only 5 occurred in introns. *ScSuSy3* had seven SNPs in the 569 bp region in each of the three species, and four of these SNPs were identical in all the three species. *ScSuSy4* had seven, nine, and nine SNPs from the above three species in the 470 bp region, respectively. Only one SNP is shared by the three species. In *ScSuSy5,* two, six, and three SNPs were found within a 577 bp region of the three respective species, but none was common among the three species(Table 3, Additional files 2 and 3).

**Table 3:** **SNP counts per base pair in the five *ScSuSy *fragments within and between the three Saccharum species**

***Saccharum *species SNP**	***ScSuSy1***	***ScSuSy2***	***ScSuSy3***	***ScSuSy4***	***ScSuSy5***	**total**	**bp/SNP**
*S. o.*	4/489	4/484	7/569	7/470	2/577	24/2589	108
*S. r.*	7/489	3/484	7/569	9/470	6/577	32/2589	81
*S. s.*	11/489	6/484	7/569	9/470	3/577	38/2589	72
Total SNPs count between the Saccharum species							
*S. o. + S. r.*	10/489	5/484	8/569	12/470	7/577	42/2589	62
*S. o. + S. s.*	14/489	9/484	10/569	15/470	5/577	53/2589	49
*S. r + S. s.*	16/489	9/484	10/569	15/470	8/577	58/2589	45
*S. s. + S. o. + S. r.*	20/489	10/484	11/569	18/470	9/577	51/2589	38
Identical SNPs count between the Saccharum species							
*S. o. \ S. r.*	2/489	2/484	6/569	4/470	1/577	15/2589	172
*S. o.\ S. s.*	1/489	1/484	4/569	1/470	0	7/2589	370
*S. s. \ S. r.*	3/489	0	4/569	3/470	1/577	11/2589	238
*S. s. \ S. o. \ S. r.*	1/489	0	4/569	1/470	0	6/2589	435

Note: *S. o.* : *S. officinarum , S. r: S. robustum, S. s:S. spontaneum.*

SNPs identified from five *ScSuSy* gene fragments were combined to estimate the SNP frequency within the genome of each species. The highest SNP frequency, at one SNP per 72 bp, is in the genome of the wild species *S. spontanuem*; the lowest SNP frequency, at one SNP per 108 bp, is in the genome of the domesticated high sugar content species *S. officinarum*. The SNP frequency in the genome of the wild species *S. robustum* is about one SNP per 81 bp, closer to that of the *S. spontaneum* (Table 3). However, pairwise DNA sequence comparison between species revealed higher SNP frequency differences. The highest SNP frequency is between the two wild species *S. robustum* and *S. spontaneum* at one SNP per 38 bp. The lowest is between the domesticated species *S. officinarum* and the wild species *S. robustum*, which share the same basic chromosome number (Table 3).

### Haplotype analysis of *ScSuSy* Genes of *Saccharum* Species

The unique combinations of SNPs in each sequenced fragment within each species were used to define haplotypes. The number of haplotypes within each gene fragments ranged from three to eight (Table 4). In *ScSuSy3*, each of the three species reached the maximum 8 haplotypes while the other four *ScSuSy* genes have varying numbers of haplotypes in the three species. When the combined haplotypes from all five gene fragments were estimated for each species, we identified 27, 35, and 32 haplotypes in LA Purple (*S. officinarum*), Molokai6081 (*S. robustum*), and SES208 (*S. spontaneum*), respectively.

**Table 4:** **Estimated number of haplotypes of SuSy genes in three *Saccharum *species**

**Haplotypes count**						
**Species**	***SuSy1***	***SuSy2***	***SuSy3***	***SuSy4***	***SuSy5***	**Summary**
*S. o* (2n = 80)	4	6	8	6	3	27
*S. r.* (2n = 80)	6	5	8	8	8	35
*S. s.* (2n = 64)	8	7	8	5	4	32
Total*	15	15	16	17	11	74*

Notes:

* Due to the consensus haplotypes among the species, the total haplotypes numbers are different from the sum of each gene.

Note: *S. o.*: *S. officinarum , S. r: S. robustum, S. s: S. spontaneum.*

We also noted consensus haplotypes among the *Saccharum ScSuSy*s genes species (Table 5). The majority of consensus haplotypes are expected to come from multiple homologous chromosomes, which are assumed to be the original haplotypes from the *Saccharum* species. The frequencies of consensus haplotypes are significantly higher than the other haplotypes (Additional file 4). In the total 75 haplotypes of the 5 *ScSuSy* genes from the three species, 16 of them are consensus haplotypes with a frequency of 52.1% of the genes fragment, which is common between at least two of the species. Obviously, there are more gene alleles from the consensus haplotypes than from the other haplotypes. Of the consensus haplotypes, 3 were present in the all three species, 1 in *ScSuSy*2 and 2 in *ScSuSy*3. These can be assumed to have existed prior to the divergence of *Saccharum* species due to it is low possibility for common haplotypes were from occasional mutational event. Pairwise comparison between *S. officinarum* and *S. spontaneum*, *S. spontaneum* and *S. robustum*, and *S. officinarum* and *S. robustum* revealed 4, 6 and 15 haplotypes that are similar to each other*.* This provides additional evidence in support of the contention that the *ScSuSy* families between *S. officinarum and S. robustum* is closer than that of the other two combinations.

**Table 5:** **Number of deduced amino acid sequences for haplotypes fragments for each SuSy genes in the *Saccharum *species**

	***ScSuSy1***	***ScSuSy2***	***ScSuSy3***	***ScSuSy4***	***ScSuSy5***	**Total**
*S. o*	1	1	4	3	3	12
*S. r.*	1	2	3	4	1	11
*S. s*	1	2	3	2	1	9
total	1	3	5	7	3	19

Notes: *The total deduced amino acid numbers are different from the sum of each gene because we presented the consensus deduced amino acid sequences among the species.

Notes: *S. o* : *S. officinarum , S. r: S. robustum, S. s: S. spontaneum.*

The corresponding amino acid sequences of each haplotype were predicted by BlastX and aligned with ClustalW (Additional file 5). Except for *ScSuSy1*, which had no non-synonymous haplotypes, there were 3, 5, 5 and 3 amino acids sequences predicted for *ScSuSy2, ScSuSy3, ScSuSy4 and ScSuSy5*, respectively. This highly suggests that multiple haplotypes results in the variation of amino acid sequence. It should be noted that the number of deduced protein sequences of haplotypes range from 1 to 7 for any of the *ScSuSy* genes of the three species, which is less than the haplotype number (Table 5). Obviously, the different haplotypes may still result in same protein sequence.

Using the information from the identification of the intron-exon boundaries for each *scSuSY* haplotype for each for each saccharum population-species, we calculated the pairwise synonymous substitutions (dS) and non-synonymous (dN) substitutions as described earlier [14]. Substitutions per synonymous site, or Ks values for each gene pairs between species were calculated using Nei-Gojobori method implemented in PAML [31]. Gene pairs giving unusually large Ks values, either because the sampled region were dissimilar or failed during the PAML calculation were discarded in the summary statistics. There were 19 pairs that meet this criteria, of which ten had Ka > = Ks. These values are affixed as Additional file 6.

## Discussion

Sugarcane was domesticated about 10,000 years ago and intensive artificial selection occurred only 100 years ago mostly on interspecific hybrids, not pure *S. officinarum* clones. Domestication, which was mainly on sugar content, might account for a small fraction of the reduced diversity in *S. officinarum* genome, but would not explain the lower diversity in *S. robustum* than in *S. spontanuem* noted in this study*.* A possible explanation might be the differential capacity among the species to produce tillers and hence biomass. Biomass, other than sucrose levels, is another noticeable contrasting trait between the three species. Natural selection for robust plants bearing more tillers led to that species to have a higher capacity for clonal propagation which consequently led to reduced diversity in *S. officinarum*, and to a lesser extent, in *S. robustum*. Plant biomass yield is highest in *S. officinarum*, then *S. robustum*, and lowest in *S. spontaneum*.

Based on phylogenetic analysis, the five *SuSy* genes from sorghum and sugarcane can be classified into the three distinct classes: *SuSy1* and *SuSy2*, *SuSy3* and *SuSy5*, and *SuSy4* clustered into Class I, II and III, respectively (Figure 3). Previous studies of *SuSy* gene family evolution in *Arabidopsis*, *Citrus* and *Populus* showed the existence of three or four distinct *SuSy* subgroups to exist in plants [25,32,33]. Interestingly, compared with the rice *SuSy* genes, the orthologous *OsSuSy3* gene is missing in sorghum. In the same manner that sequence comparison of rice and sorghum revealed about 7% of the genes appear to be unique to sorghum [9], the *OsSuSy3* could be from lineage specific gene duplication event in rice after its divergence from the ancestor of sorghum and sugarcane.

The occurrence of the first *SuSy* gene duplication event was predicted to be before the angiosperm/gymnosperm divergence which occurred about 200 mya; and a later duplication of SuSys within subclasses among angiosperms must have arisen before the separation of the monocots and dicots, which is thought to have occurred about 140–150 MYA [34]. The results of the phylogenetic analysis of this study are consistent with the timeline described above. In addition, the predicted molecular weights of the 5 polypeptides are close, ranging from 91.71 to 98.79 kDa; and among them, *ScSuSy*1-4 are around 93 kDa, which is consistent with the SDS-PAGE results [21].

The average SNP frequency of *ScSuSy* genes in the three species is lower than one per 58 bp in the *S. officinarum*, one per 35 bp in the sugarcane hybrid cultivar Q165 [35], and an average of one every 50 bp as occurs by the EST estimation [36]. Based on the SNP frequencies of the *Saccarhum* species, the predicted SNP frequencies of hybrids between *S.officinarum* (LA Purple) and *S. spontaneum* (SES208) is about 1 SNP per 50 bp; this is still higher than the SNP frequencies (one every 35 based ) of sugarcane cultivar Q165. This could be the result of purifying selection in *ScSuSy*, a primary gene family in sucrose metabolism, hence reduce genetic diversity [37].

Since sugarcane is an autopolyploid with each locus having multiple haplotypes from eight or more depending on the ploidy level of the accession. This multiple haplotypes per gene, an indication of heterozygosity level, is likely to have contributed to the high biomass yield of sugarcane. However, there are indications that the increased fixation of elite alleles in modern breeding germplasm is already inhibiting further genetic gain of sugarcane. As modern sugarcane cultivars are derived from crosses between *S. officinarum*, *S. spontaneum,* as well as *S. robustum*, analyses of haplotypes and allele complexity of genes in sucrose metabolism in domesticated and wild species will improve our understanding of genetic basis for sucrose accumulation in modern sugarcane cultivars and the level of heterozygosity within the genome of each species.

The haplotype diversity can be seen as an indication of heterozygosity level of both genes and species. All of the *ScSuSy* genes, except perhaps *ScSuSy5*, showed relatively high levels of heterozygosity (Table 4). It is possible, however, that the short fragment length and random distribution of SNPs, the haplotype number of *ScSuSy5* might be only less variable within the length of fragments used for the haplotype analysis. The five *ScSuSy* family members were evolved before the divergence between sugarcane and sorghum 8 MYA (Figure 1), whereas haplotype diversity in *Saccharum* occurred after the WGD events less than 1.5 MYA. There is no correlation between *ScSuSy* family members and haplotype diversity.

SNP frequency does not correlate to haplotype diversity or protein diversity. Among the three species, *S. robustum* has the most haplotypes (Table 4), not *S. spontaneum* that has the highest SNP frequency. Moreover, *S. officinarum*, which has the lowest SNP frequency, has the highest number of deduced protein sequences (Table 5), whereas *S. spontaneum*, which has the highest SNP frequency, has the lowest number of deduced protein sequences. A pairwise dS/dN ratio test for selection (Table 5) showed that 10 out of the 19 pairs had Ka > =Ks; an indication of positive selection. Thus, SNP differences between species could have been the results of positive selection towards accumulation of sucrose in the high sugar content *S. officinarum* and intermediate sugar content in *S. robustum*. Detailed examination of haplotype diversity revealed that the difference of haplotype numbers between the two wild species *S. robustum* and *S. spontaneum* is from *ScSuSy5* with the maximum of eight haplotypes in *S. robustum* and four haplotypes in *S. spontaneum*. No transcript from this gene was detected in the five tissue types in all three species. It is not clear whether this gene has a function in sugar metabolism. The analysis of haplotypes provides the opportunity to infer the evolutionary history of a DNA region [38,39]. In this study, the consensus haplotypes for the *ScSuSy* genes in *Saccharum* species could be used for estimating the origin of haplotypes and discovering the relation among the *Saccharum* species. The number of consensus haplotypes between *S. officinarum* and *S. robustum* is significantly higher (t-test, P< 0.05) than the other two combinations of the three species, which reinforce the notion that the divergence between these two species occurred after their common ancestor diverged from *S. spontaneum*[12,40,41]. A total of 94 haplotypes in 74 unique haplotypes are present in the 1,366 fragments of *SuSy* genes (Table 4). Of 1,366 fragments, 726 sequences in 17 unique haplotypes are common among the three species. As the three species in the study are octoploid, the haplotypes of the five *SuSy* genes of species results from the 5 groups of 24 homologous chromosomes. The consensus 17 unique haplotypes, which occur at a frequency of 53.1% (726/1366), are derived from half of the homologous chromosomes. The frequencies of the consensus haplotypes are much higher than any species specific haplotypes, suggesting that the consensus haplotypes were derived from multiple homologous chromosomes. These results reflect the fact that the brief evolutionary history of haplotypes accounts for only a fraction of the time since the divergence of the five *ScSuSy* gene members. Selection constraint on these genes in the sucrose biosynthesis and degradation further reduced the diversification of haplotypes.

The SNP frequency within each species and the number of haplotypes within each genome provide crucial information for assessing strategies to sequence these complex genomes. Each homologous chromosome consists of a mosaic of haplotypes sharing various degree of sequence identity with haplotypes in any of the other seven chromosomes. With a SNP frequency at one per 108 bp or higher, it is not possible to have a consensus sequence among eight homologous chromosomes. There is no diploid or tetraploid accessions in *Saccharum*, and simplest genome is a tetraploid (haploid) accession of *S. spontaneum* SES 208 generated by anther culture [42]. This genotype would be the best material for sequencing the first genome of *Saccharum*, and even for that tetraploid genome, ultra long sequence reads from single molecules are needed for correct assembling of the homologous chromosomes and annotation of allelic variations with haplotypes varying from three to eight in homologous regions.

## Conclusions

Analyses of SNP and haplotypes in three primary *Saccharum* species revealed insights into the level of heterozygosity within each octoploid genome and the evolutionary history of these three genomes. The within genome heterozygosity as measured by SNP frequency is the lowest in the domesticated species *S. officinarum* and highest in the wild species *S. spontaneum*, suggesting that the WGD events occurred earlier in *S. spontaneum* than in *S. officinarum*. *S. officinarum* shared more common SNPs with *S, robustum* than with S. spontaneum, confirming the closer phylogenetic relationship between *S. officinarum* and *S. robustum*. This may also explain the success of integrating disease/pest resistance genes from *S. spontaneum* as these two species contain more diverse sets of R genes than between *S. officinarum* and *S. robustum*. Although the number of haplotypes is fewer in *S. officinarum* than in *S. spontaneum*, the number of deduced protein sequences is higher in *S. officinarum* than in *S. spontaneum*, a sign of positive selection on these *ScSuSy* genes in the high sugar content species *S. officinarum*.

## Methods

### Plant materials

Three varieties of *Saccharum* species were used in the study: *S. officinarum* LA Purple (2n = 8× = 80), *S. robustum* Molokai 6081 (2n = 8× = 80), and *S. spontaneum* SES208 (2n = 8× = 64) [3]. Genomic DNA from young leaf tissues for each of the three accessions were isolated using Qiagen DNeasy miniprep kit following the manufacturer’s protocol (Qiagen, Inc., Valencia, CA, USA).

### Database Searches and gene Annotation for the *SuSy* genes in sorghum

Six *Arabidopsis SuSy* sequences (At1G73370, AT1G73370, AT5G2083, At5g49190, At5g37180, and At4g02280) and six rice *SuSy* sequences [24], to identify the full set of *SuSy* genes in the sorghum (*Sorghum bicolor*) genome. BLASTn and tBLASTn search (http://blast.ncbi.nlm.nih.gov/) hits that has similarity scores of >50.0 and probability scores of <10^-4^ were retained for further analysis. Wherever possible, we checked the published annotations of the sorghum genomic clones against full-length cDNA clones and ESTs from sugarcane and sorghum in NCBI. We also checked the predicted amino acid sequences against the conserved motifs of SuSy. For genomic sequences that had not previously been annotated, we supplemented the above methods with the use of Genscan (http://genes.mit.edu/GENSCAN.html)[43] and FGENESH (http://linux1.softberry.com/berry.phtml) gene prediction software. *SuSy* protein sequences were analyzed using tools available at http://us.expasy.org/tools/.

### Verification of *SuSy* genes members in sugarcane

The genomic and predicted mRNA sequences of sorghum were used to search the ESTs database of sugarcane. Based on the sorghum *SuSy* genes, the ESTs hits of sugarcane were classified for predicting the number the *SuSy* genes in sugarcane. PCR primers were designed, using Primer Premier c5.00, from the sorghum genome and sugarcane EST sequences to amplify an approximately 500nt, intron-containing region in the *Saccharum* genome (Additional file 7). PCR reactions were carried out in a total of 50 μl volume containing 30 ng template DNA, 0.2 μM of each PCR primer and 25 μl 2 × GoTaq® Green Master Mix (Promega, WI, USA). PCR conditions were: 3 min at 94°C followed by 35 cycles of 10 s at 94°C, 30 s at the appropriate annealing temperature (50-65°C), 30 s at 72°C and an additional extension step of 6 min at 72°C. PCR amplification was verified by running samples out on a 1% agarose gel.

The primers for genomic amplification were also used for verifying *SuSy* transcripts by RT-PCR (Additional file 7). Using TRIzol® (Invitrogen, USA), total RNA was extracted from two different stem, mature leaf and leaf roll of three *Saccharum* species. The total RNA was treated with RNase-free DNase I(Ambion, TX,USA) and reverse transcribed using ImProm-II™ Reverse Transcription System (Promega, WI, USA). RT-PCR reactions were carried out as previously described.

In addition, using the predicted sorghum *SuSy* cDNA sequence as a reference, a cDNA database from Illumina RNA-seq sequencing with 42 million pair-end reads were searched by NOVOALIGN with default parameters (http://www.novocraft.com/main/index.php). The sequences of target genes from NOVOALIGN results were obtained using Tablet software [44] and assembled by Sequencher 4.0 (Minimum Match Percentage 96%, Minimum overlap 20%) to achieve the full cDNA sequences of the *SuSy* genes. The corresponding amino acid sequences of the *SuSy* genes were deduced through BLASTx and ORF finder (http://www.ncbi.nlm.nih.gov/gorf/gorf.html).

### Phylogenetic dendrogram of *SuSy* gene family members

The amino acid sequences of *SuSy* gene family members including members from angiosperms, gymnosperms and bacteria, were identified by searching public databases available at NCBI (http://www.ncbi.nlm.nih.gov). A phylogenetic dendrogram of *SuSy* members was made using the deduced amino acid sequences by ClustalW v2 program (http://www.ebi.ac.uk/Tools/msa/clustalw2/). The tree view program version 1.6.6 [45] was used to generate unrooted trees with the stability of the tree obtained estimated by bootstrap analysis for 100 replications.

### Identification of SNPs and Haplotype Analysis of the sc*SuSy* Gene in *Saccarhum* species

We cloned the gene fragments following the modified procedure done to characterize the SPS III in sugarcane [29]. Each DNA samples from three *Saccharum* species was amplified twice for the *SuSy* genes. The resulting amplified PCR products were then purified using Wizard® SV Gel and PCR Clean-Up System (Promega, WI, USA) cloned into pGEMT Easy Vector (Promega, WI, USA) and transformed into chemically competent E. coli strain JM109. Individual colonies were grown in LB media at 37C overnight, and plasmids were isolated using a modified alkaline lysis procedure. The purified fragments were sequenced using the T7 primer and the big dye terminator cycle sequencing kit (Applied Biosystems) performed by the W.M. Keck Center for Comparative and Functional Genomics, University of Illinois at Urbana-Champaign ( http://www.biotech.uiuc.edu/). To avoid confounding error from PCR recombination and sequencing, only SNPs and haplotypes that were observed in at least two colonies were considered for sequencing.

The generated sequence reads were inspected and trimmed manually for quality using Sequencher 4.10.1. The sequence reads of each of the *sbSuSy* gene fragment for each saccharum species were separately aligned to identify the *sbSuSy* SNPs and SNPs haplotypes within species. Conversely, all the *SuSy* gene sequences were aligned together to investigate SNP polymorphism between the three saccharum species.

The unique combinations of SNPs found after alignment were used to define haplotypes for each *scSusy* gene per species. Assessment of haplotype SNP counts and SNP frequency were assessed manually. From these alignments as well, the protein sequences were predicted by BlastX and aligned with ClustalW [46], converted back to DNA (codon) alignments with PAL2NAL [47] from which synonymous substitutions (dS) and non-synonymous substitutions (dN) was calculated following the same pipeline as described in an earlier report [14].

## Competing interests

The authors declare that they have no competing interests.

## Authors’ contributions

JZ and RM conceived the study and designed the experiments. JZ carried out the experiments and analyzed the data. JZ and RM wrote the manuscript. JA and YC analyzed the data and contributed to the writing of the manuscript. All authors read and approved the final paper.

## Supplementary Material

Additional file 1 List of sucrose synthase gene sequences used in this study. Click here for file

Additional file 2 **The SNPs frequencies in the 5 SuSy genes of Three *Saccharum *Species.** Click here for file

Additional file 3 **SNP position within the SuSy haplotype fragment of the *Saccharum *species.** Click here for file

Additional file 4 **Summary of the estimated haplotype analysis of SuSy genes in three *Saccharum *species.** Click here for file

Additional file 5 **The predicted amino acid of the haplotypes of SuSy genes fragments from the *Saccharum *species.** Click here for file

Additional file 6 Summary of Ka, Ks calculation. Click here for file

Additional file 7 The primers for genes verification and haplotype analysis. Click here for file
