# Supplementary material for: Haplotype analysis of sucrose synthase gene family in three Saccharum species
Source: BMC Genomics. 2013 May 10;14:314. doi: 10.1186/1471-2164-14-314 (PMC3668173; doi:10.1186/1471-2164-14-314)
Supplement: Additional file 3 — SNP position within the SuSy haplotype fragment of the Saccharum species. [file 1471-2164-14-314-S3.ppt]

## Slide 1
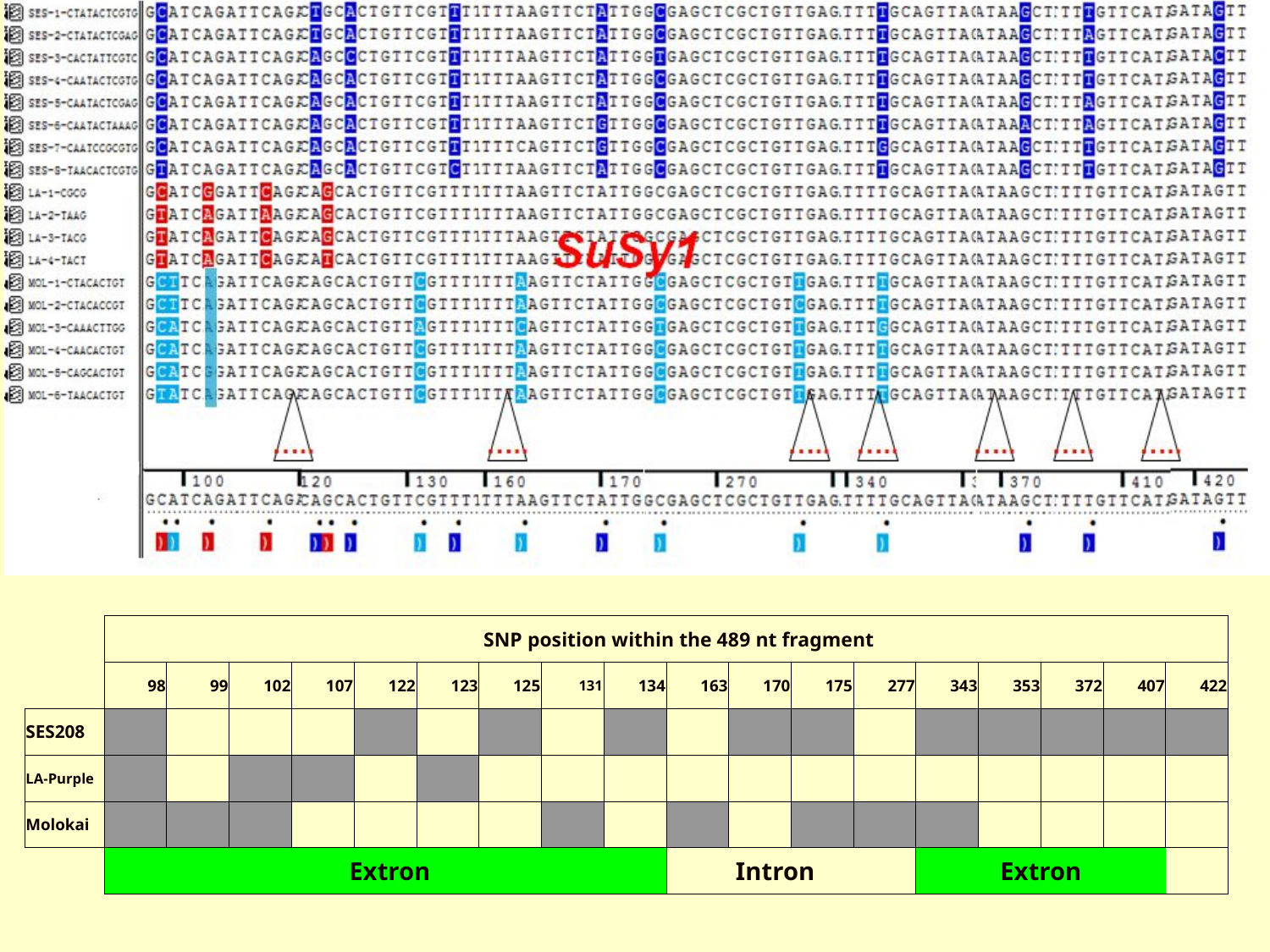

| | SNP position within the 489 nt fragment | | | | | | | | | | | | | | | | | |
| --- | --- | --- | --- | --- | --- | --- | --- | --- | --- | --- | --- | --- | --- | --- | --- | --- | --- | --- |
| | 98 | 99 | 102 | 107 | 122 | 123 | 125 | 131 | 134 | 163 | 170 | 175 | 277 | 343 | 353 | 372 | 407 | 422 |
| SES208 | | | | | | | | | | | | | | | | | | |
| LA-Purple | | | | | | | | | | | | | | | | | | |
| Molokai | | | | | | | | | | | | | | | | | | |
| | Extron | | | | | | | | | Intron | | | | Extron | | | | |

## Slide 2
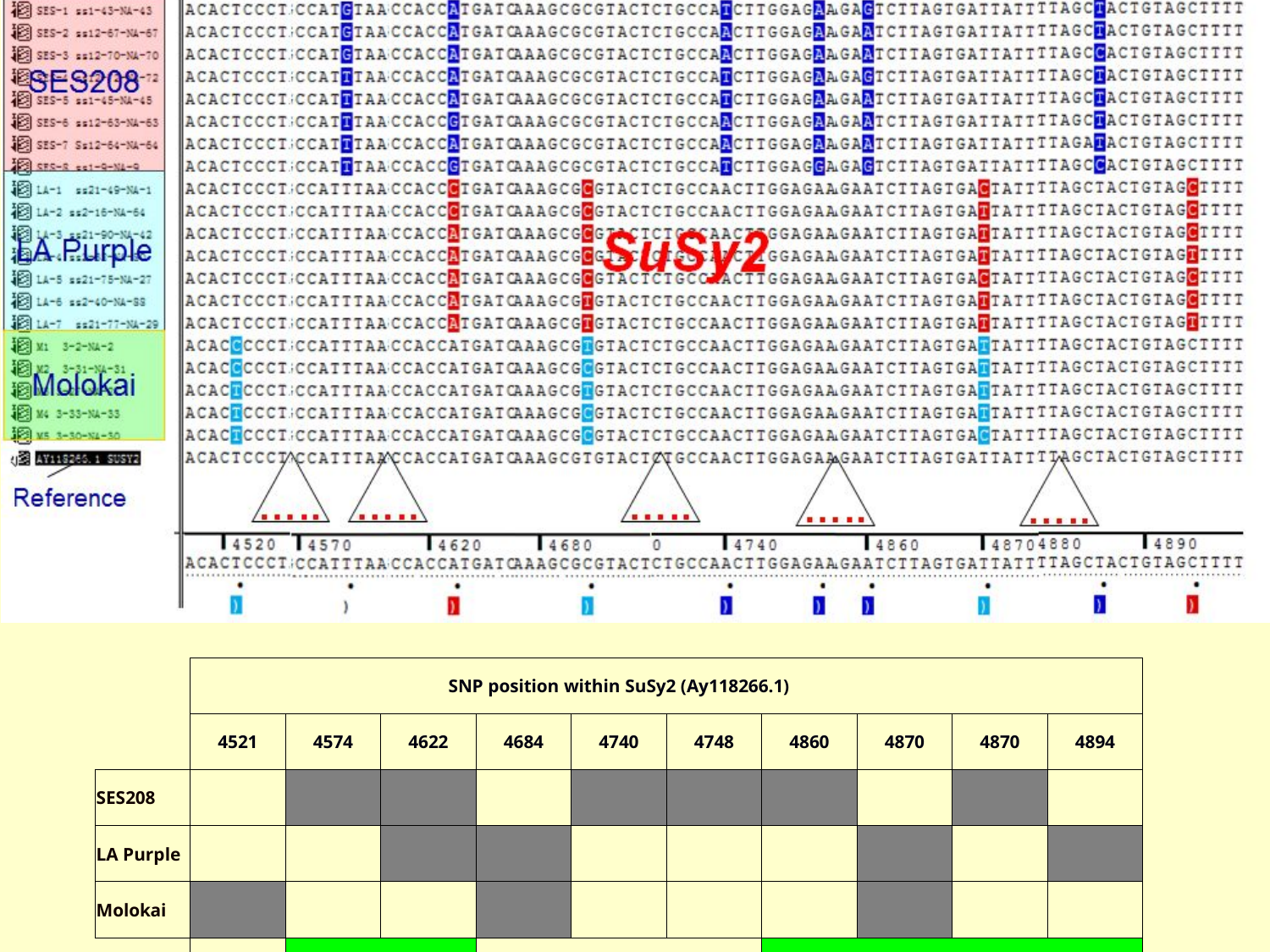

| | SNP position within SuSy2 (Ay118266.1) | | | | | | | | | |
| --- | --- | --- | --- | --- | --- | --- | --- | --- | --- | --- |
| | 4521 | 4574 | 4622 | 4684 | 4740 | 4748 | 4860 | 4870 | 4870 | 4894 |
| SES208 | | | | | | | | | | |
| LA Purple | | | | | | | | | | |
| Molokai | | | | | | | | | | |
| | | Extron | | Intron | | | Extron | | | |

## Slide 3
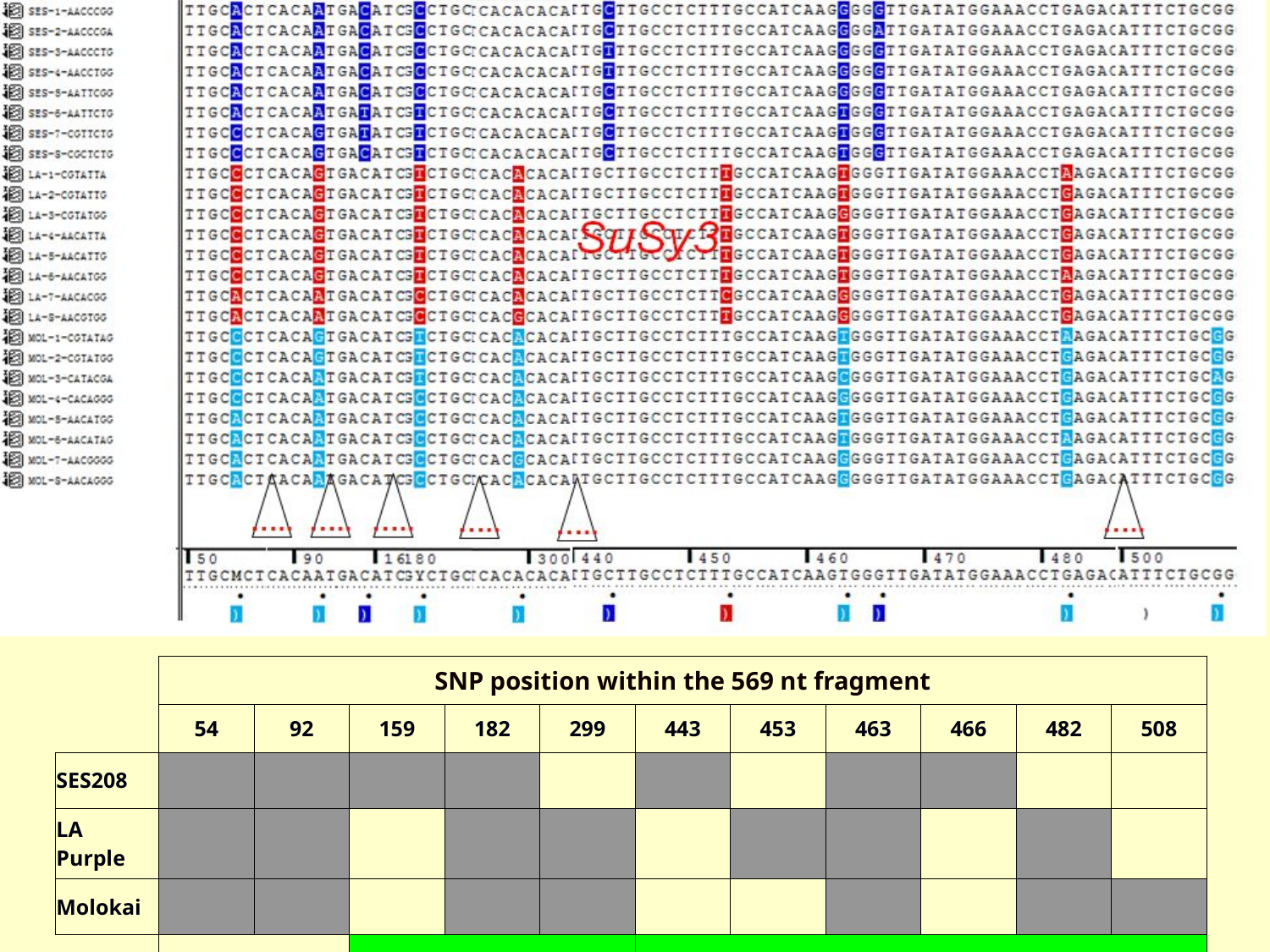

| | SNP position within the 569 nt fragment | | | | | | | | | | |
| --- | --- | --- | --- | --- | --- | --- | --- | --- | --- | --- | --- |
| | 54 | 92 | 159 | 182 | 299 | 443 | 453 | 463 | 466 | 482 | 508 |
| SES208 | | | | | | | | | | | |
| LA Purple | | | | | | | | | | | |
| Molokai | | | | | | | | | | | |
| | Intron | | Extron | | | Extron | | | | | |

## Slide 4
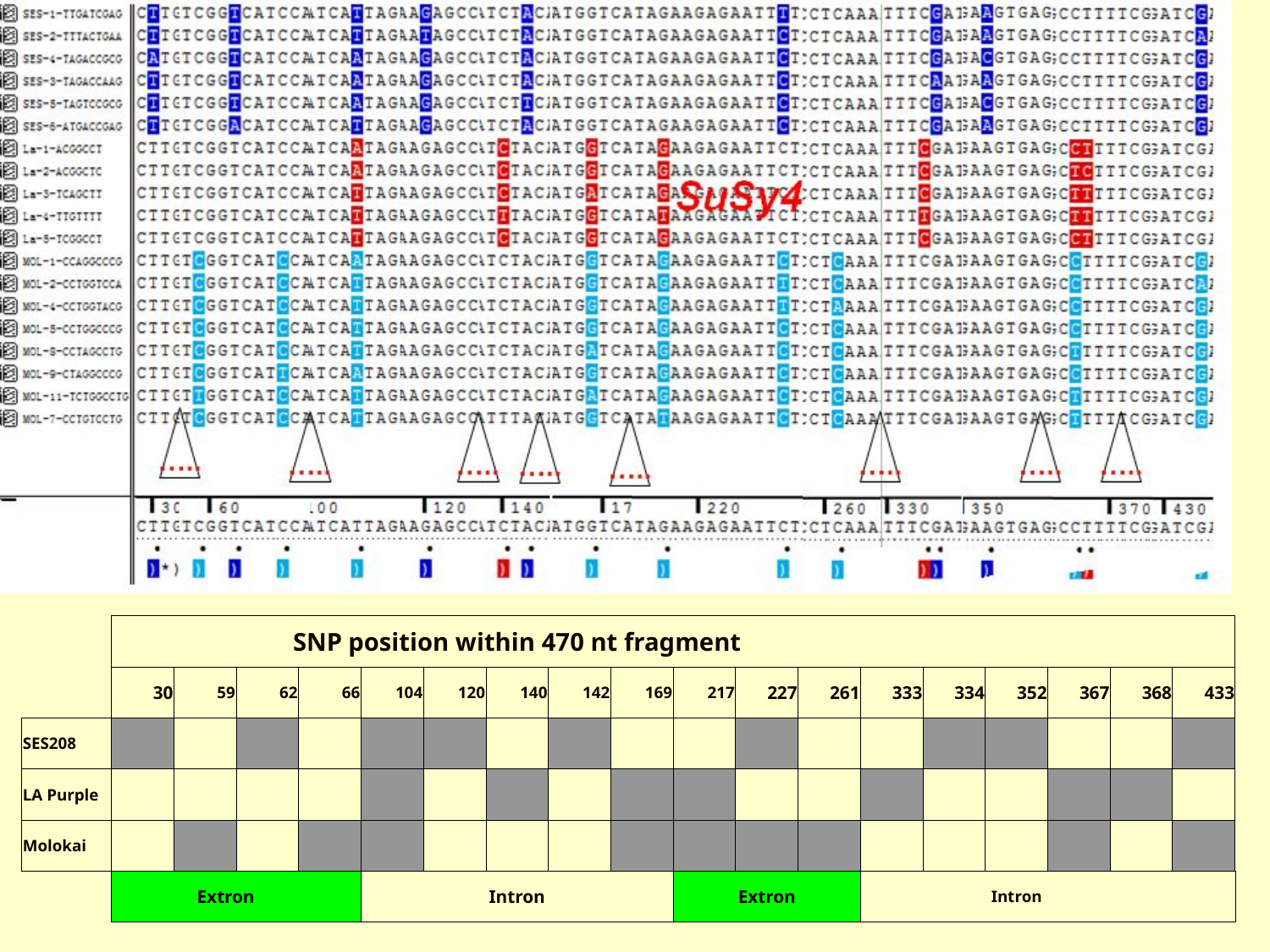

| | | | SNP position within 470 nt fragment | | | | | | | | | | | | | | | |
| --- | --- | --- | --- | --- | --- | --- | --- | --- | --- | --- | --- | --- | --- | --- | --- | --- | --- | --- |
| | 30 | 59 | 62 | 66 | 104 | 120 | 140 | 142 | 169 | 217 | 227 | 261 | 333 | 334 | 352 | 367 | 368 | 433 |
| SES208 | | | | | | | | | | | | | | | | | | |
| LA Purple | | | | | | | | | | | | | | | | | | |
| Molokai | | | | | | | | | | | | | | | | | | |
| | Extron | | | | | Intron | | | | Extron | | | Intron | | | | | |

## Slide 5
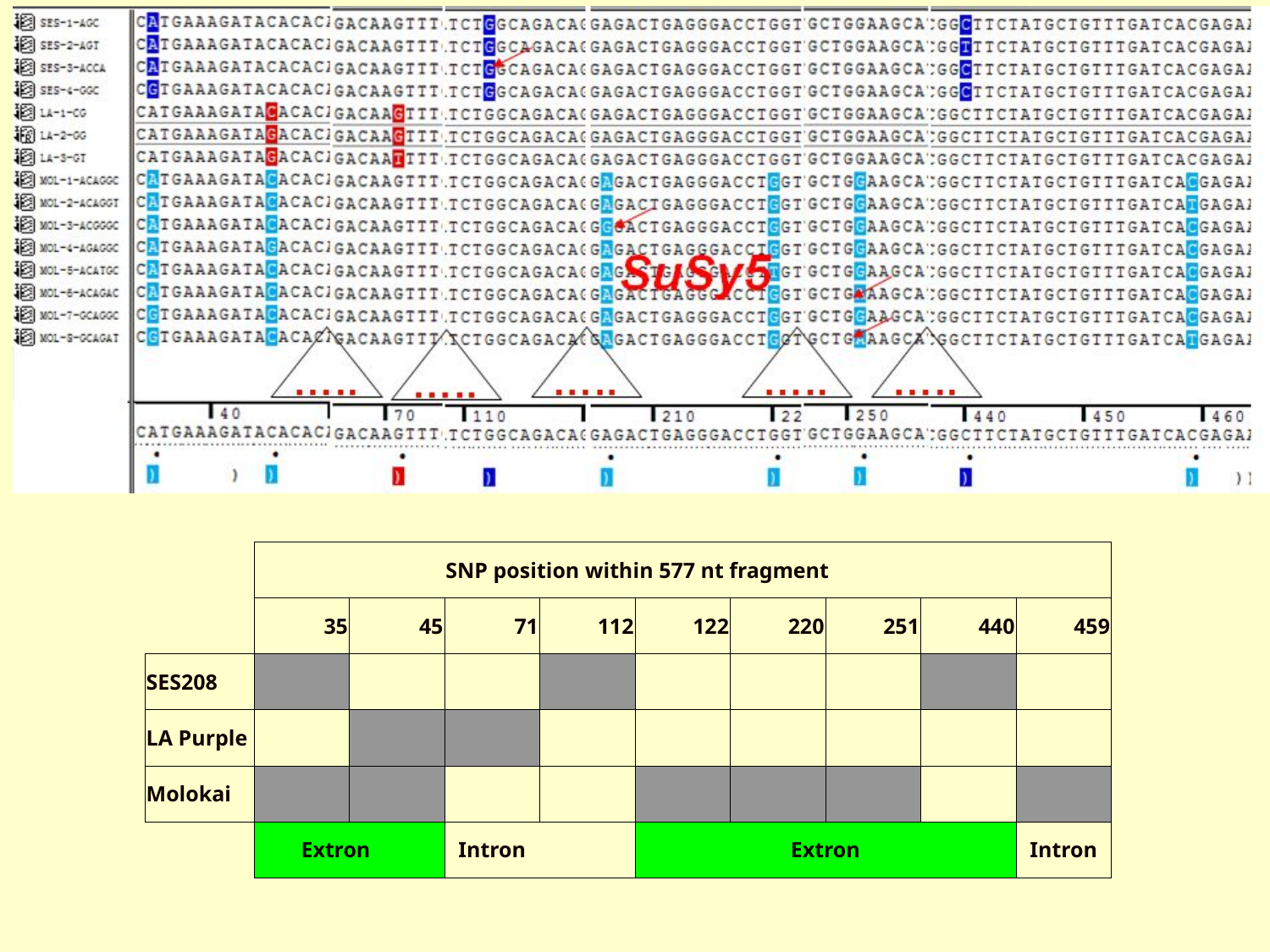

| | | | SNP position within 577 nt fragment | | | | | | |
| --- | --- | --- | --- | --- | --- | --- | --- | --- | --- |
| | 35 | 45 | 71 | 112 | 122 | 220 | 251 | 440 | 459 |
| SES208 | | | | | | | | | |
| LA Purple | | | | | | | | | |
| Molokai | | | | | | | | | |
| | Extron | | Intron | | Extron | | | | Intron |
