# Supplementary material for: Haplotype analysis of sucrose synthase gene family in three Saccharum species
Source: BMC Genomics. 2013 May 10;14:314. doi: 10.1186/1471-2164-14-314 (PMC3668173; doi:10.1186/1471-2164-14-314)
Supplement: Additional file 4 — Summary of the estimated haplotype analysis of SuSy genes in three Saccharum species. [file 1471-2164-14-314-S4.docx]

Additional file 4.Summary of the estimated haplotype analysis of SuSy genes in three Saccharum species

| 1.Summary of the estimated haplotype analysis of SuSy1 in three Saccharum species | | |  |  |
| --- | --- | --- | --- | --- |
|  |  | haplotypes(polymorphism numbers in 489 bps) | HP | Percentage |
| S. spontaneum (SES208)  (119 fragments) | SES-SS1-1 | C..A..A..C..T..G..A..C..T..A..A..C..T..T..C..G..T..G.. | 6 | 5.04% |
|  | SES-SS1-2 | C..A..A..C..T..G..A..C..T..A..A..C..T..T..C..G..A..G.. | 3 | 2.52% |
|  | SES-SS1-3 | C..A..A..C..A..G..C..C..T..A..A..T..T..T..C..G..T..C.. | 12 | 10.08% |
|  | SES-SS1-4(A) | C..A..A..C..A..G..A..C..T..A..A..C..T..T..C..G..T..G.. | 36 | 30.25% |
|  | SES-SS1-5 | C..A..A..C..A..G..A..C..T..A..A..C..T..T..C..G..A..G.. | 11 | 9.24% |
|  | SES-SS1-6 | C..A..A..C..A..G..A..C..T..A..G..C..T..T..A..A..A..G.. | 12 | 10.08% |
|  | SES-SS1-7 | C..A..A..C..A..G..A..C..T..C..G..C..T..G..C..G..T..G.. | 9 | 7.56% |
|  | SES-SS1-8 | T..A..A..C..A..G..A..C..C..A..A..C..T..T..C..G..T..G.. | 12 | 10.08% |
|  |  | potential PCR recombination | 18 | 15.13% |
| *S.officinarum*  (LA-Purple)  (98 fragments) | LA-SS1-1(B) | C..A..G..C..A..G..A..C..T..A..A..C..T..T..C..G..T..G.. | 64 | 65.31% |
|  | LA-SS1-2 | T..A..A..A..A..G..A..C..T..A..A..C..T..T..C..G..T..G.. | 10 | 10.20% |
|  | LA-SS1-3(C) | T..A..A..C..A..G..A..C..T..A..A..C..T..T..C..G..T..G.. | 13 | 13.27% |
|  | LA-SS1-4 | T..A..A..C..A..T..A..C..T..A..A..C..T..T..C..G..T..G.. | 10 | 10.20% |
|  |  | potential PCR recombination | 1 | 1.02% |
| *S. robustum*  (Moloka-6081)  (70 fragments) | MOL-SS1-1 | C..T..A..C..A..G..A..C..T..A..A..C..T..T..C..G..T..G.. | 4 | 5.71% |
|  | MOL-SS1-2 | C..T..A..C..A..G..A..C..T..A..A..C..C..T..C..G..T..G.. | 8 | 11.43% |
|  | MOL-SS1-3 | C..A..A..C..A..G..A..A..T..C..A..T..T..G..C..G..T..G.. | 3 | 4.29% |
|  | MOL-SS1-4(A) | C..A..A..C..A..G..A..C..T..A..A..C..T..T..C..G..T..G.. | 4 | 5.71% |
|  | MOL-SS1-5(B) | C..A..G..C..A..G..A..C..T..A..A..C..T..T..C..G..T..G.. | 19 | 27.14% |
|  | MOL-SS1-6(C) | T..A..A..C..A..G..A..C..T..A..A..C..T..T..C..G..T..G.. | 21 | 30.00% |
|  |  | potential PCR recombination | 11 | 15.71% |
|  |  |  |  |  |

| 2.Summary of the estimated haplotype analysis of SuSy2 in three Saccharum species | | |  |  |
| --- | --- | --- | --- | --- |
|  | Haplotype name | haplotypes(polymorphism numbers in 484 bps) | haplotype frequencies | Percentage |
| S. spontaneum (SES208)  (85 fragments) | SES-SS2-1 | T..G..A..C..T..A..G..T..T..C.. | 6 | 7.06% |
|  | SES-SS2-2 | T..G..A..C..A..A..A..T..T..C.. | 18 | 21.18% |
|  | SES-SS2-3 | T..T..A..C..T..A..G..T..T..C.. | 27 | 31.76% |
|  | SES-SS2-4 | T..T..A..C..T..A..A..T..T..C.. | 3 | 3.53% |
|  | SES-SS2-5 | T..T..A..C..T..G..G..T..C..C.. | 5 | 5.88% |
|  | SES-SS2-6(A) | T..T..A..C..A..A..A..T..T..C.. | 13 | 15.29% |
|  | SES-SS2-7 | T..T..G..C..A..A..A..T..T..C.. | 5 | 5.88% |
|  | SES-SS2-8 | potential PCR recombination | 8 | 9.41% |
| S.officinarum  (LA-Purple)  (82 fragments) | LA-SS2-1 | T..T..C..C..A..A..A..C..T..C.. | 2 | 2.44% |
|  | LA-SS2-2 | T..T..C..C..A..A..A..T..T..C.. | 3 | 3.66% |
|  | LA-SS2-3(A) | T..T..A..C..A..A..A..T..T..C.. | 38 | 46.34% |
|  | LA-SS2-4 | T..T..A..C..A..A..A..T..T..T.. | 7 | 8.54% |
|  | LA-SS2-5(B) | T..T..A..C..A..A..A..C..T..C.. | 14 | 17.07% |
|  | LA-SS2-6(C) | T..T..A..T..A..A..A..T..T..C.. | 17 | 20.73% |
|  | LA-SS2-7 | potential PCR recombination | 1 | 1.22% |
| *S. robustum*  (Moloka-6081)  (82 fragments) | MOL-SS2-1 | C..T..A..T..A..A..A..T..T..C.. | 3 | 3.66% |
|  | MOL-SS2-2 | C..T..A..C..A..A..A..T..T..C.. | 3 | 3.66% |
|  | MOL-SS2-3(C) | T..T..A..T..A..A..A..T..T..C.. | 10 | 12.20% |
|  | MOL-SS2-4(A) | T..T..A..C..A..A..A..T..T..C.. | 57 | 69.51% |
|  | MOL-SS2-5(B) | T..T..A..C..A..A..A..C..T..C.. | 9 | 10.98% |
|  | MOL-SS2-6 | potential PCR recombination | 0 | 0.00% |
|  |  |  |  |  |

| 3.Summary of the estimated haplotype analysis of SuSy3 in three Saccharum species | | | |  |  |
| --- | --- | --- | --- | --- | --- |
|  | |  | haplotypes(polymorphism numbers in 484 bps) | HP | Percentage |
| S. spontaneum (SES208)  (115 fragments) | | SES-SS3-1 | A..A..C..C..A..C..T..G..G..G..G.. | 60 | 52.17% |
|  |  | SES -SS3-2 | A..A..C..C..A..C..T..G..A..G..G.. | 6 | 5.22% |
|  |  | SES -SS3-3(D) | A..A..C..C..A..C..T..T..G..G..G.. | 6 | 5.22% |
|  |  | SES -SS3-4 | A..A..C..C..A..T..T..G..G..G..G.. | 13 | 11.30% |
|  |  | SES -SS3-5 | A..A..T..T..A..C..T..G..G..G..G.. | 2 | 1.74% |
|  |  | SES -SS3-6 | A..A..T..T..A..C..T..T..G..G..G.. | 4 | 3.48% |
|  |  | SES -SS3-7 | C..G..T..T..A..C..T..T..G..G..G.. | 16 | 13.91% |
|  |  | SES -SS3-8(B) | C..G..C..T..A..C..T..T..G..G..G.. | 2 | 1.74% |
| potential PCR recombination | | |  | 6 | 5.22% |
| S.officinarum  (LA-Purple)  (98 fragments) | | LA-SS3-1(A) | C..G..C..T..A..C..T..T..G..A..G.. | 20 | 20.41% |
|  |  | LA -SS3-2(B) | C..G..C..T..A..C..T..T..G..G..G.. | 36 | 36.73% |
|  |  | LA -SS3-3 | C..G..C..T..A..C..T..G..G..G..G.. | 4 | 4.08% |
|  |  | LA -SS3-4(C) | A..A..C..C..A..C..T..T..G..A..G.. | 5 | 5.10% |
|  |  | LA -SS3-5(D) | A..A..C..C..A..C..T..T..G..G..G.. | 5 | 5.10% |
|  |  | LA -SS3-6(E) | A..A..C..C..A..C..T..G..G..G..G.. | 4 | 4.08% |
|  |  | LA -SS3-7 | A..A..C..C..A..C..C..G..G..G..G.. | 4 | 4.08% |
|  |  | LA -SS3-8(F) | A..A..C..C..G..C..T..G..G..G..G.. | 7 | 7.14% |
| potential PCR recombination | | | 13 | 13.27% |  |
| *S. robustum*  (Moloka-6081)  ( 77 fragments) | | MOL-SS3-1(A) | C..G..C..T..A..C..T..T..G..A..G.. | 8 | 10.39% |
|  |  | MOL-SS3-2(B) | C..G..C..T..A..C..T..T..G..G..G.. | 28 | 36.36% |
|  |  | MOL-SS3-3 | C..A..C..T..A..C..T..C..G..G..A.. | 4 | 5.19% |
|  |  | MOL-SS3-4 | C..A..C..C..A..C..T..G..G..G..G.. | 4 | 5.19% |
|  |  | MOL-SS3-5(D) | A..A..C..C..A..C..T..T..G..G..G.. | 5 | 6.49% |
|  |  | MOL-SS3-6(C) | A..A..C..C..A..C..T..T..G..A..G.. | 3 | 3.90% |
|  |  | MOL-SS3-7(F) | A..A..C..C..G..C..T..G..G..G..G.. | 3 | 3.90% |
|  |  | MOL-SS3-8(E) | A..A..C..C..A..C..T..G..G..G..G.. | 11 | 14.29% |
|  | |  | potential PCR recombination | 11 | 14.29% |
|  | |  |  |  |  |
| 4.Summary of the estimated haplotype analysis of SuSy4 in three Saccharum species | | | |  |  |
|  | |  | haplotypes(polymorphism numbers in 484 bps) | HP | Percentage |
| *S. spontaneum* (SES208)  (90 fragments ) | | SES-SS4-1 | T..C..T..C..G..A..C..T..G..G..C..C..C..G..A..C..T..G.. | 25 | 27.78% |
|  |  | SES-SS4-2 | T..C..T..C..T..A..C..C..G..G..T..C..C..G..A..C..T..A.. | 6 | 6.67% |
|  |  | SES-SS4-3 | T..C..A..C..G..A..C..C..G..G..C..C..C..A..A..C..T..G.. | 15 | 16.67% |
|  |  | SES-SS4-4 | T..C..A..C..G..A..C..C..G..G..C..C..C..G..C..C..T..G.. | 14 | 15.56% |
|  |  | SES-SS4-5 | T..C..A..C..G..T..C..C..G..G..C..C..C..G..C..C..T..G.. | 12 | 13.33% |
|  |  | SES-SS4-6 | A..C..T..C..G..A..C..C..G..G..C..C..C..G..A..C..T..G.. | 8 | 8.89% |
| potential PCR recombination | | |  | 6 | 7.41% |
| S.officinarum  (LA-Purple)  (81 fragments) | LA-SS4-1(A) | | T..C..T..C..A..G..C..A..G..G..C..C..C..G..A..C..T..G.. | 29 | 31.18% |
|  | LA-SS4-2 | | T..C..T..C..A..G..C..A..G..G..C..C..C..G..A..T..C..G.. | 2 | 2.15% |
|  | LA-SS4-3 | | T..C..T..C..T..G..C..A..A..G..C..C..C..G..A..T..T..G.. | 45 | 48.39% |
|  | LA-SS4-4 | | T..C..T..C..T..G..T..A..G..T..C..C..T..G..A..T..T..G.. | 8 | 8.60% |
|  | LA-SS4-5(B) | | T..C..T..C..T..G..C..A..G..G..C..C..C..G..A..C..T..G.. | 3 | 3.23% |
|  | potential PCR recombination | |  | 10 | 11.11% |
|  |  |  |  |  |  |
| *S. robustum*  (Moloka-6081)  ( 95 fragments) | MOL-SS4-1(A) | | T..C..T..C..A..G..C..A..G..G..C..C..C..G..A..C..T..G.. | 21 | 22.11% |
|  | MOL-SS4-2 | | T..C..T..C..T..G..C..A..G..G..T..C..C..G..A..C..T..A.. | 10 | 10.53% |
|  | MOL-SS4-3 | | T..C..T..C..T..G..C..A..G..G..T..A..C..G..A..C..T..G.. | 8 | 8.42% |
|  | MOL-SS4-4(B) | | T..C..T..C..T..G..C..A..G..G..C..C..C..G..A..C..T..G.. | 9 | 9.47% |
|  | MOL-SS4-5 | | T..C..T..C..T..G..C..A..G..T..C..C..C..G..A..T..T..G.. | 8 | 8.42% |
|  | MOL-SS4-6 | | T..C..T..C..T..G..C..A..A..G..C..C..C..G..A..T..T..G.. | 10 | 10.53% |
|  | MOL-SS4-7 | | T..C..T..C..T..G..C..A..A..G..C..C..C..G..A..T..T..G.. | 11 | 11.58% |
|  | MOL-SS4-8 | | T..T..T..C..T..G..C..A..A..G..C..C..C..G..A..T..T..G.. | 10 | 10.53% |
|  |  | | potential PCR recombination | 8 | 8.42% |
| consensus |  | | T..C..T..C..T..G..C..A..G..G..C..C..C..G..A..C..T..G.. |  |  |
|  |  | |  |  |  |
| 5.Summary of the estimated haplotype analysis of SuSy5 in three Saccharum species | | | |  |  |
|  | |  | haplotypes(polymorphism numbers in 484 bps) | haplotype frequencies | percentage |
| S. spontaneum (SES208)  (87 fragments) | | SES-SS5-1(A) | A..C..G..G..A..G..G..C..C.. | 50 | 57.47% |
|  |  | SES-SS5-2 | A..C..G..G..A..G..G..T..C.. | 7 | 8.05% |
|  |  | SES-SS5-3 | A..C..G..C..A..G..G..C..C.. | 18 | 20.69% |
|  |  | SES-SS5-4(B) | G..C..G..G..A..G..G..C..C.. | 10 | 11.49% |
|  |  |  | potential PCR recombination | 2 | 2.30% |
| S.officinarum  (LA-Purple)  (87 fragments) | | LA-SS5-1(A) | A..C..G..G..A..G..G..C..C.. | 53 | 60.92% |
|  |  | LA-SS5-2(C) | A..G..G..G..A..G..G..C..C.. | 24 | 27.59% |
|  |  | LA-SS5-3 | A..G..T..G..A..G..G..C..C.. | 9 | 10.34% |
|  |  |  | potential PCR recombination | 1 | 1.15% |
| *S. robustum*  (Moloka-6081)  (88 fragments) | | MOL-SS5-1(A) | A..C..G..G..A..G..G..C..C.. | 41 | 46.59% |
|  |  | MOL-SS5-2 | A..C..G..G..A..G..G..C..T.. | 2 | 2.27% |
|  |  | MOL-SS5-3 | A..C..G..G..G..G..G..C..C.. | 2 | 2.27% |
|  |  | MOL-SS5-4(C) | A..G..G..G..A..G..G..C..C.. | 6 | 6.82% |
|  |  | MOL-SS5-5 | A..C..G..G..A..T..G..C..C.. | 5 | 5.68% |
|  |  | MOL-SS5-6 | A..C..G..G..A..G..A..C..C.. | 2 | 2.27% |
|  |  | MOL-SS5-7(B) | G..C..G..G..A..G..G..C..C.. | 22 | 25.00% |
|  |  | MOL-SS5-8 | G..C..G..G..A..G..A..C..T.. | 5 | 5.68% |
|  | |  | potential PCR recombination | 3 | 3.41% |
| consensus | |  | A..C..G..G..A..G..G..C..C.. |  |  |

Notes: The consensus haplotype were colored with same.
