# Supplementary material for: Haplotype analysis of sucrose synthase gene family in three Saccharum species
Source: BMC Genomics. 2013 May 10;14:314. doi: 10.1186/1471-2164-14-314 (PMC3668173; doi:10.1186/1471-2164-14-314)
Supplement: Additional file 5 — The predicted amino acid of the haplotypes of SuSy genes fragments from the Saccharum species. [file 1471-2164-14-314-S5.docx]

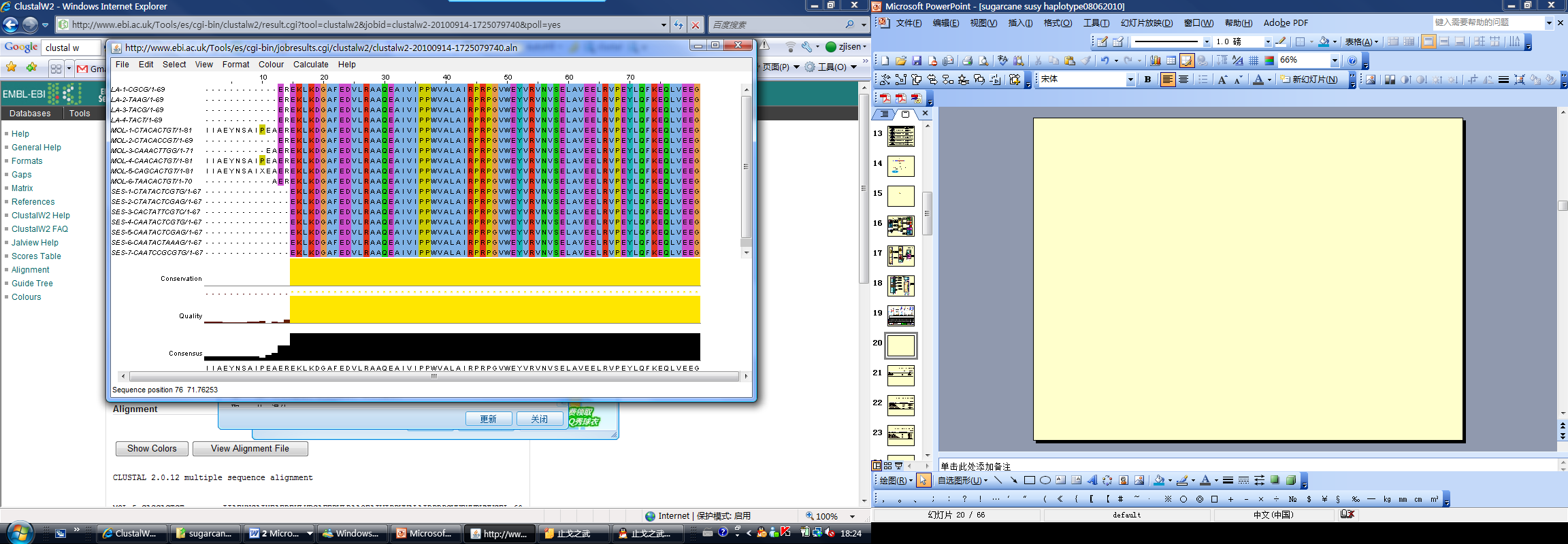
1. The Predicted Amino Acid of the Haplotypes of SuSy1 Fragment from the *Saccharum* Species


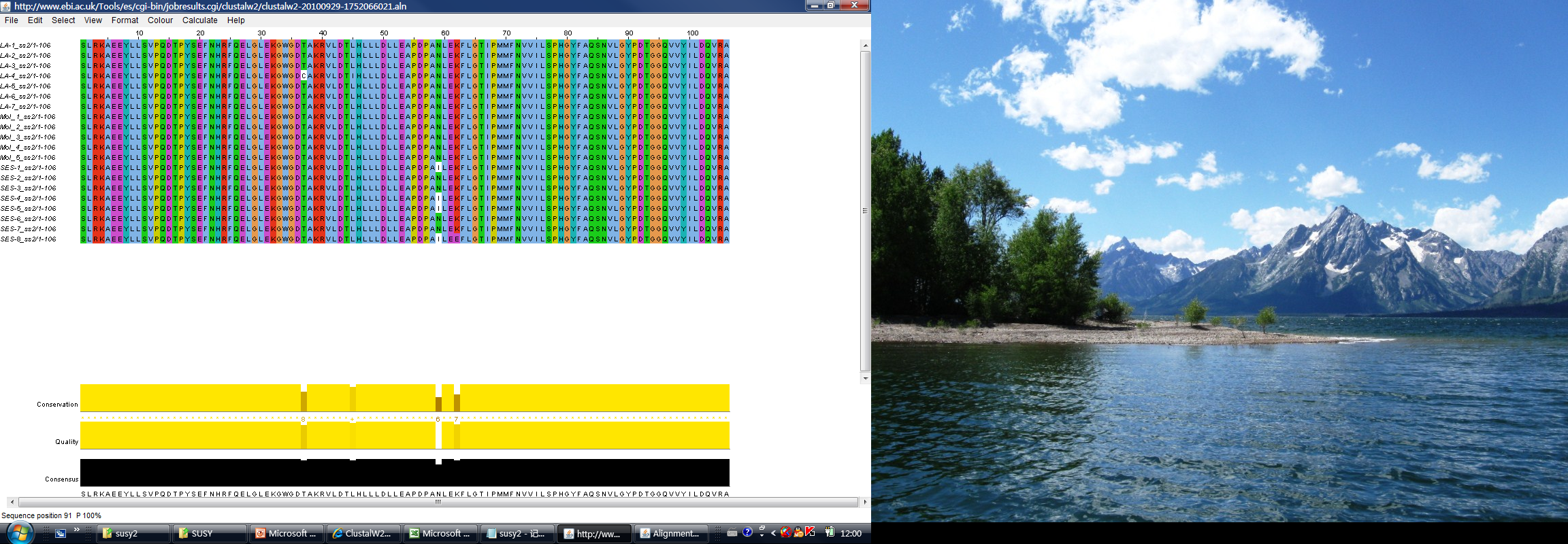


2. The Predicted Amino Acid of the Haplotypes of SuSy2 Fragment from the *Saccharum* Species


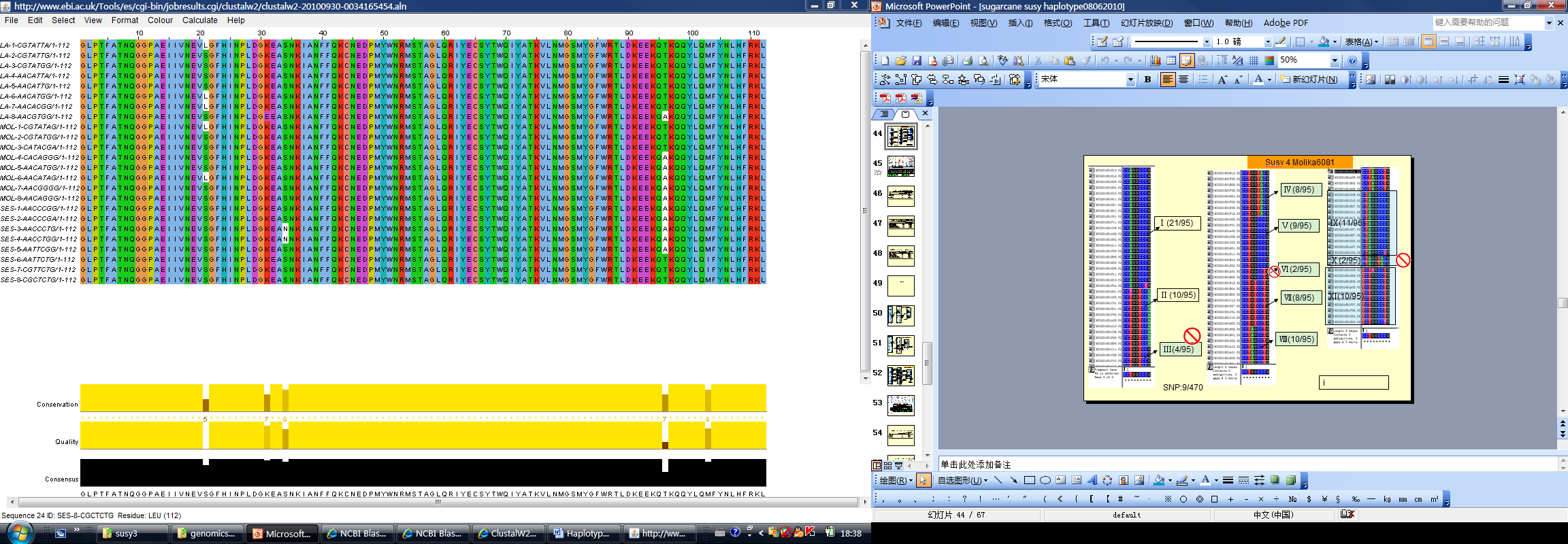


3. The Predicted Amino Acid of the Haplotypes of SuSy3 Fragment from the *Saccharum* Species


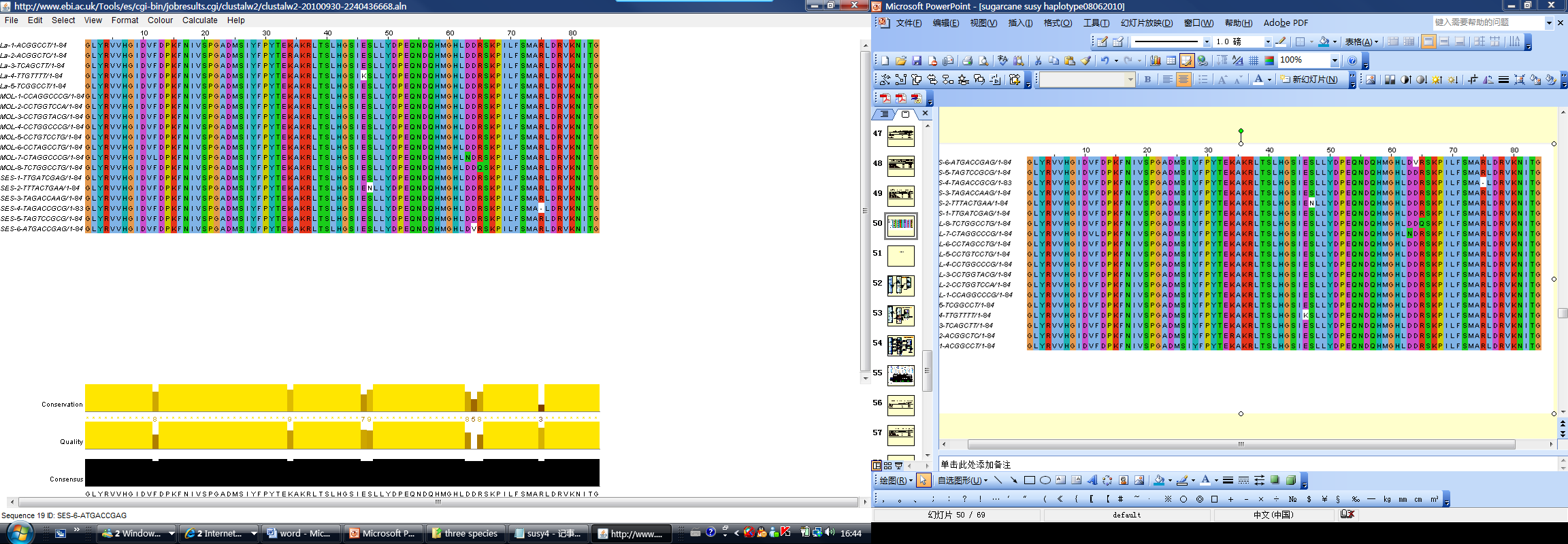


4.The Predicted Amino Acid of the Haplotypes of SuSy4 Fragment from the *Saccharum* Species


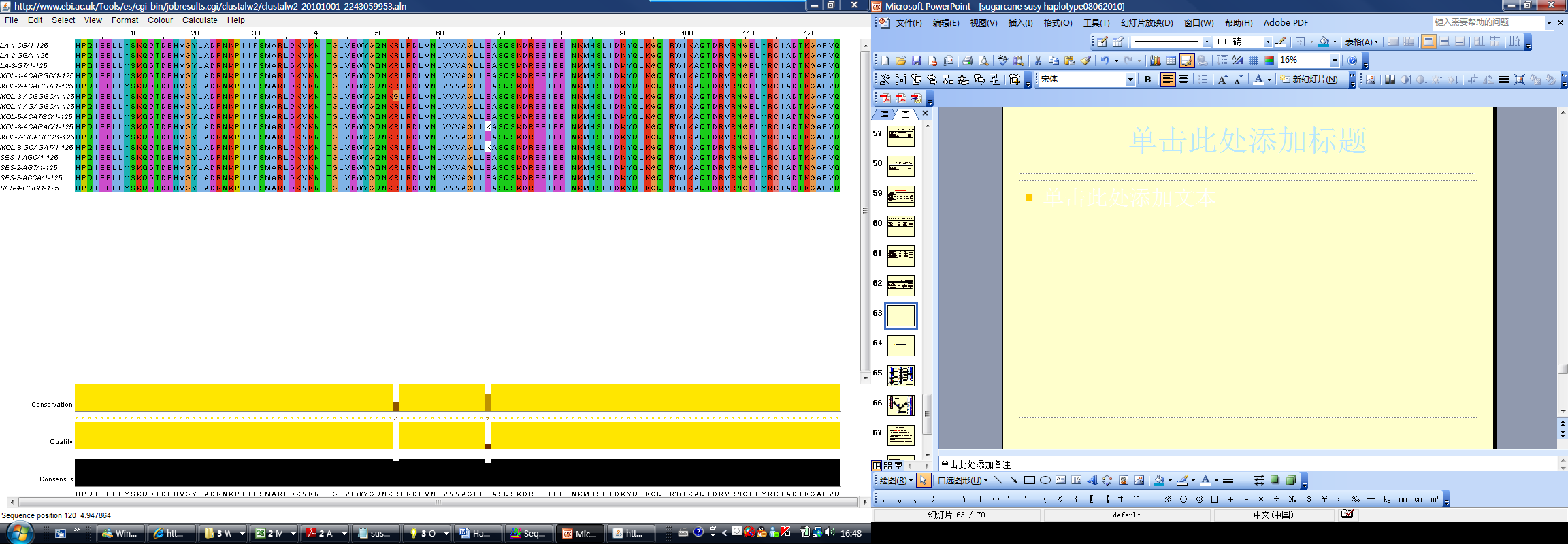


5. The Predicted Amino Acid of the Haplotypes of SuSy5 Fragment from the *Saccharum* Species
