# Supplementary material for: Haplotype analysis of sucrose synthase gene family in three Saccharum species
Source: BMC Genomics. 2013 May 10;14:314. doi: 10.1186/1471-2164-14-314 (PMC3668173; doi:10.1186/1471-2164-14-314)
Supplement: Additional file 6 — Summary of Ka, Ks calculation. [file 1471-2164-14-314-S6.doc]

The primers for genes verification and haplotype analysis

| Gene name | Forward | Reverse | Amplicons | Exons |
| --- | --- | --- | --- | --- |
| *ScSuSy1* | GATCATTGCCGAGTACAACA | TCAAAGTCCAGCTCAAGAACA | 489 | 279 |
| *ScSuSy2* | TCATCCCTTAGAAAGGCAGAA | AGCACGGACTTGATCCAAAA | 486 | 319 |
| *ScSuSy3* | ACTGTGGGCTGCCAACCTTT | CCACTTTCGGCACCGCATTT | 545 | 358 |
| *ScSuSy4* | CTGGACTGTACCGCGTTGTC | TCCTGTTATGTTCTTCACCCT | 470 | 253 |
| *ScSuSy5* | ACCACAGCAGGCATTAGATT | CACCCACAGATTGAGGAGTT | 577 | 256 |
